# From Viscount Knutsford

**Published:** 1918-07-20

**Authors:** 

**Affiliations:** London Hospital, Whitechapel, E.


### From Viscount Knutsford.

SIR,—In consequence of the challenge in Dr. Chapple's letter, I am obliged to ask you to allow me to state that I do deny the truth of his statements, and also the deductions from the small portion of them which is true.—Yours faithfully, KNUTSFORD.

London Hospital, Whitechapel, E., July 16.

